# Online information for parents caring for their premature baby at home: A focus group study and systematic web search

**DOI:** 10.1111/hex.12670

**Published:** 2018-01-30

**Authors:** Fiona Alderdice, Phyl Gargan, Emma McCall, Linda Franck

**Affiliations:** ^1^ Queen's University Belfast Belfast UK; ^2^ University of Oxford England UK; ^3^ University of California San Francisco San Francisco CA USA

**Keywords:** focus groups, parents, post‐discharge, premature, web search

## Abstract

**Background:**

Online resources are a source of information for parents of premature babies when their baby is discharged from hospital.

**Objectives:**

To explore what topics parents deemed important after returning home from hospital with their premature baby and to evaluate the quality of existing websites that provide information for parents post‐discharge.

**Methods:**

In stage 1, 23 parents living in Northern Ireland participated in three focus groups and shared their information and support needs following the discharge of their infant(s). In stage 2, a World Wide Web (WWW) search was conducted using Google, Yahoo and Bing search engines. Websites meeting pre‐specified inclusion criteria were reviewed using two website assessment tools and by calculating a readability score. Website content was compared to the topics identified by parents in the focus groups.

**Results:**

Five overarching topics were identified across the three focus groups: life at home after neonatal care, taking care of our family, taking care of our premature baby, baby's growth and development and help with getting support and advice. Twenty‐nine sites were identified that met the systematic web search inclusion criteria. Fifteen (52%) covered all five topics identified by parents to some extent and 9 (31%) provided current, accurate and relevant information based on the assessment criteria.

**Conclusion:**

Parents reported the need for information and support post‐discharge from hospital. This was not always available to them, and relevant online resources were of varying quality. Listening to parents needs and preferences can facilitate the development of high‐quality, evidence‐based, parent‐centred resources.

## 1 BACKGROUND

Going home from hospital can be a stressful time for parents who are faced with looking after their baby in the absence of professional support for the first time.^1^ Awareness of infant needs is described as a learning process utilizing external resources, trial‐and‐error and an internal intuitive sense, particularly in identifying and dealing with infant pain/discomfort^2^ and with communicating with health‐care professionals.^3^ Existing research suggests that parents are apprehensive about their infants’ perceived fragile health, losing the support of the neonatal team, their parenting skills and ability to perform caregiving procedures.^1^, ^3^

A recent initiative involving parents and health professionals in the United Kingdom asked participants to prioritize what research should be conducted on prematurity. After interventions to prevent preterm birth, preventing infection and necrotizing enterocolitis, and the best treatment for lung damage, the most important priority identified by parents and clinicians was “What should be included in packages of care to support parents and families or carers when a premature baby is discharged from hospital”.^4^ Once at home, preterm infant care is often multifaceted involving medical follow‐up, liaison with health visitors, allied health professionals and voluntary support such as family support volunteer services. Families of preterm infants need appropriate, concise information to complement that delivered by their service providers that can be readily accessed when and where they need it.

One way to achieve this is through the provision of mobile device‐friendly social technology and web‐based content, which are increasingly being used in health care for patient information and support.^5^ Gabbert et al^6^ surveyed 141 parents of very low birthweight infants in Germany and found all respondents said they used the Internet with 80% using it at least once every day. When searching for information during the stay in the neonatal intensive care unit (NICU) and after discharge, most parents used Google search engines. The most frequently searched topics were “specific medical problems associated with prematurity,” “general information on prematurity” and “outcome of preterm infants”. While parents considered the Internet a useful source of information on prematurity after discharge home, only 20% felt that their questions had been fully answered online.^6^

Therefore, establishing ways of assuring the availability of high‐quality evidence‐based health information is an important step to enhance consumer confidence. Eysenbach et al^7^ conducted a systematic review of empirical studies assessing the quality of health information for consumers on the Internet. Seventy‐nine studies met the review inclusion criteria, but these were heterogeneous due to the different methodologies, rigour and information quality assessment criteria. Consequently, the ability to draw firm conclusions was limited and the authors recommended the need to develop definitions of quality criteria to increase the robustness of systematic assessment of websites. More recently, a number of measures have evolved to facilitate the standardization of this process although to date, there is no consensus as to a gold standard assessment.

There is a demand for web‐based information to help fill the gaps in information and support that parents experience when they go home with their premature baby. This research was conducted to inform the development of a web resource for parents of premature babies when they go home. We also wanted to ascertain the quality of available information on the Internet, to assess whether or not the content matches the needs of parents and whether or not a new web resource was warranted. Therefore, the overall aims of this study were

1. To explore with parents, using focus groups, what topics they deemed important for their parenting role after returning home from hospital with their premature baby**.**
2. To evaluate existing websites on the quality of the information provided using quality assessment tools for Internet resources.
3. To identify whether the information topics described by parents in the focus groups are addressed on existing websites.

## 2 METHODS

### 2.1 Methods stage 1: Focus groups with parents

#### 2.1.1 Design

Focus group methodology using content analysis, was used to encourage the generation of ideas and expression of shared experiences and common viewpoints within a group of peers. The number of focus groups was based on the resources available and the recommendation that for simple research questions three to four focus groups are required.^8^

#### 2.1.2 Participants

Three focus groups were conducted in three different locations in Northern Ireland. A total of 23 parents took part in a focus group (10 in focus group 1, eight in focus group 2 and five in focus group 3). Parents who had a premature baby (less than 37 weeks completed gestational age) discharged from hospital were eligible to participate. Those unable to adequately understand verbal explanations in English or who had special communication needs were excluded.

#### 2.1.3 Process

Eligible participants were identified by TinyLife, the Northern Ireland premature and sick baby charity, through parents on their mailing list and through social media (TinyLife Facebook page). Facebook and email were the main communication mechanisms used to stay in touch with parents who had been in contact with the charity. TinyLife has 30,000 Facebook followers, 1,100 families their database and 13 parent support groups who receive monthly communication. An invitation letter was sent by email and posted on Facebook. Those interested in participating in the research study notified the TinyLife key contact of their interest. The TinyLife key contact co‐ordinated the best time and location for parents and referred the details on to the research team.

The focus groups were conducted in April 2016 in three locations reflecting city and rural areas and using a neutral venue routinely used by TinyLife for their local parent support groups. We collected data from groups in different regions to capture variation and breadth of experiences. When parents arrived, they provided informed consent prior to completing a short questionnaire on demographic information, health information about their preterm baby and some basic information on their NICU experience. The questionnaire data were used strictly for descriptive statistical analysis of the sample of participants in order to provide context. The group ground rules were discussed to highlight confidentiality and to facilitate open discussions and parents and researchers introduced themselves to one another. Two researchers were always present, one to facilitate and one to take notes and to co‐ordinate group activity. Participants were asked a series of open‐ended questions (see Appendix 1 for the focus group schedule, which was developed with the project advisory group made up of researchers, health professionals and parent representatives). In addition, notes were taken on a flip chart under the four focus group questions so parents could monitor comments. The flipchart notes generated during the focus group were used by each parent to identify their top five priorities for a website by placing a sticky dot beside their five most important topic areas. Two parents had to leave early and were unable to complete the dot exercise. Each session lasted between approximately 45‐90 minutes. Sessions were audio‐recorded for later transcription. Participants were given travel expenses and £20 towards child care cover.

#### 2.1.4 Qualitative analyses

The focus group audio tapes were professionally transcribed verbatim, cross‐referenced by the researcher and analysed by hand along with field notes and flip charts. The first stage of the content analyses was conducted during the focus group with topics being written on a flipchart as the topic was raised. Data analysis of the transcripts was undertaken using a conventional content analysis approach, as described by Morse and Field.^9^ Initially, the transcripts were read and re‐read by two members of the core research team (FA and LF), to ensure familiarization of the data. Topics were identified by highlighting segments of data, which were coded by identifying persistent words, phrases or concepts. Data were then grouped according to topic, allowing further identification of subtopic. Following coding, the data were categorized to reflect the overall sense of the topic and the relationships between the categories. These were then compared with the topic notes on the flipchart to ensure consistency and to identify any gaps. Related categories were then merged into an overarching topic, where appropriate. All steps of the qualitative analyses were conducted by two members of the team (FA and LF).

#### 2.1.5 Ethical considerations

Fundamental principles of good research practice including informed consent, voluntary participation, and confidentiality and data protection procedures were applied as a minimum standard. No personal identifiers were placed on the focus group data or questionnaires. Transcripts were de‐identified, and audio recordings were destroyed after transcripts were checked for accuracy.

The research governance procedures for Queen's University Belfast, including the Code of Conduct and Integrity in Research, were followed and ethical approval sought from Lancaster NRES (IRAS project id: 187383).

### 2.2 Methods stage 2: Systematic web search

#### 2.2.1 Search process

To identify relevant websites, a search of the World Wide Web (WWW) was conducted on a university computer using the three top search engines that are currently used by web users in the UK: Google, Yahoo and Bing (June 2016 http://www.ebizmba.com/articles/search-engines ).

The following search terms were used

- Going home after NICU
- Life after NICU
- Advice parents after NICU
- Support parents premature baby
- Care parents premature baby.

These terms were selected to provide a mix of general prematurity websites and specific sites on premature babies at home to ensure we covered a broad base and reflected words used by parents. The final set of terms was agreed with the project advisory group. The first 25 websites identified for each key phrase in each of the three search engine comprising a total of 375 websites and their hyperlinks were assessed for inclusion criteria in the review. The searches were conducted between the 23 May 16 and the 30 June 16. The decision to assess the top 25 websites was based on (i) other web searches have found that on average, after the first 17 hits, relevant hits were found to have been listed previously^10^ and (ii) Internet searchers rarely look beyond the first 20 search results.^11^

Websites were only included in the review if they met the following inclusion criteria:

Publically available, written in English, written for parents/guardians of premature babies and information pertinent to after discharge.

#### 2.2.2 Exclusion criteria

Websites designed for health professionals and websites that are solely blogs, links to documents (e.g PDFs) or videos.

#### 2.2.3 Procedure

The screening of the websites was conducted independently by two members of the research team (PG and FA) using the predefined inclusion and exclusion criteria. Where there was disagreement or uncertainty about a website meeting inclusion criteria (n = 8), consensus was agreed and arbitrated by a third member of the research team (LF). If the website met the inclusion criteria, the website was reviewed using the CLEAR and CRAAP tests. The first five websites were scored by two authors (PG and FA), and as there was no major discrepancy in score, the rest were scored by PG with any uncertainties (n = 4 websites) being discussed and the score agreed with FA. The Flesch‐Kincaid readability score was calculated by PG.

We chose two website assessment tools for use in this review one developed for consumers (CLEAR^12^) and one for academics (CRAAP tool^13^) to explore similarities and differences in assessment. The CLEAR tool was developed primarily to aid NICU parents in deciphering online information as they search for information pertaining to the health of their infants.^12^ The tool covers five criteria: Current (how current is the website?), Language (how understandable is the language used?), Easy (how well organized is it?), Author (who wrote the information?) and Reason (does the website state its purpose?). The first four criteria have a maximum score of 3 and the fifth criterion has a maximum score of 2. Four points are deducted from the total score if the website was selling a product or had commercial advertisements on the site. The maximum overall score is 14. A score of 12 or more is considered excellent/credible, a score of 7‐11 indicates that the reader should look for further information elsewhere, and a score of 0‐6 is rated poor, indicating that the reader should disregard the site and look elsewhere for information.

The CRAAP test^13^ was developed by Librarians at the California State University, Chico, and has five criteria to determine whether information is credible or not. The five criteria are Currency (how up to date is the information?), Relevance (Does the information answer your question?), Authority (who is the author?), Accuracy (where does the information come from?) and Purpose (is the information there to inform, teach, sell or persuade?). Each criterion is scored on a scale from 1 (worst) to 10 (best possible) with a maximum score of 50. Scores between 45‐50 were rated “excellent,” 40‐44 “good,” 35‐39 “average” and 30‐34 “borderline acceptable” and below 30 “unacceptable.”

In addition, a Flesch–Kincaid readability score was assigned^14^ for the text on the opening page of each website. The Flesch‐Kincaid reading ease score is designed to indicate how difficult a reading passage in English is to understand. The first paragraph of the website was put into a Word document and Flesch 2.0 for windows was used to assess the text for reading age A reading ease score of around 65 is a good target for most business writing, 70‐80 is easily understood by 11‐ to 12‐year‐olds, and 80‐90 is perceived to be conversational English for consumers.

Finally, the content of each website was examined to see whether there was evidence that the topics identified by parents in the stage 1 focus groups were included. Two members of the research team (PG and FA) independently looked at the website in detail exploring all sections for reference to the topics generated by parents. If the website made reference to the topic but not beyond a few sentences, this was identified as “to some extent.” If a whole section or subsection was identified, this was considered to be “covered well.”

## 3 RESULTS OF THE STAGE 1 FOCUS GROUP STUDY

The characteristics of participating parents and their preterm baby (first born in the case of multiple births) can be found in Table 1. The majority of participants were mothers living with their partners and approximately half had other children. Two fathers participated: one with their partner present and one alone. Participants had babies with a broad range of gestational ages and birthweights. Parents who had experienced multiple births and had babies with ongoing health problems were also represented.

**Table 1:** Characteristics of parents who participated in the focus group (n = 23)

Parent and baby characteristics	
Number (%) Women	21 (91)
Mean (SD) Age in Years	33.8 (5.9)
Number (%) Living with partner	21 (91)
Number (%) Had other children	12 (52)
Number (%) Singleton	14 (61)
Number (%) Twins	7 (30)
Number (%) 3 or more	2 (9)
Mean (SD) Gestational age at birth in weeks (first child)	30.8 (3.97)
Mean (SD) Birthweight in kg	1.650 (0.620)
Mean (SD) Age Baby is Now in months	17.7 (15.77)
Baby Health	
No problem	10 (44)
Needed additional care/follow‐up	11 (48)
Died	1 (4)
Data missing	1 (4)

**Figure 1:**
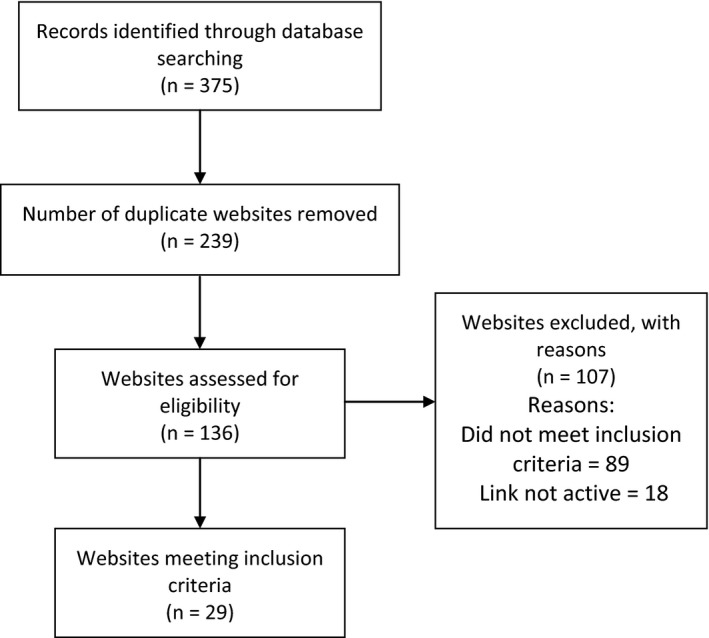
Flow chart of identification of websites that met the inclusion criteria

Table 2 identifies the priority areas identified by parents during the focus groups when they were asked to place five dots beside the topics that they considered to be most important. Eight topics were identified related to content: advice on parent self‐care, infant development, getting support and advice from others, life at home after neonatal care, worry about our baby getting sick again, feeding and digestion issues, help with communicating with health professionals and general advice on caring for our baby. A further topic related to web information was also identified. No further topics were identified during the content analysis of the focus group transcripts. Example quotations reflecting each topic are provided in Table 2. Parents were asked to rank the topics identified by their group, all but two topics were highlighted as priorities in all focus groups; “General advice on caring for our baby” and “Help with communicating with health professionals” were not top priority areas in Focus Group 3 (the smallest focus group with 5 parents participating).

**Table 2:** Priority topics identified by parents

Priority	Number (dot exercise) n = 110	Example quotations
Advice on parent self‐care Subtopics: Physical health Emotional health Relationships	22	*“Get some sleep before your baby is home” “Don't put too much pressure on yourself” FG1* *“I maybe didn't feel upbeat even when things were smooth and progressing as I was still trying to process everything” FG2* *“It mightn't be just as soon as you come home but I remember the first time my husband and I decided to go somewhere after we came home…I think it's important to do something” FG2*
Infant development Subtopics: Comparing to term babies When to worry	15	*“It's hard not to think about developmental milestones and compare what they are doing even though you shouldn't; my sister in law is a health visitor and she was able to give me some idea of when they might start smiling and then looking at their hands etc. but just don't get too caught up in it” FG3* *“All babies are different” FG2* *“Again it's that thing of knowing when should I worry? What should I be expecting him to achieve or not achieve?” FG2*
Getting Support and Advice from others Subtopics: Other parents of premature babies Wider family and friends	14	*“Get in touch with other people who have been through the same experience” FG1* *“If you are not coping don't be afraid to ask for help, let someone else step in — it's not a big deal to let someone else help you” FG3*
Life at home after neonatal care Subtopics: Ready to go home Stressed Need for routine	12	*“I was able to go home and be a mummy and I was so ready for that” FG 2* *“It is not always instinctive about what you should do with your baby — even though other people say it is” FG1* *“I just came home really stressed — jumping out of a sleep to check if he was still breathing; I was also concerned about the strength of the medication and they had shown us how to accurately measure it so you were always afraid of giving him the tiniest drop extra, and being a first time mother added to it” FG2*
Worry about our baby getting sick again Subtopic: Germs and infection Controlling our environment	11	*“I was most worried about infection, I was just not prepared for how I was going to feel when the house was coming down with visitors; it had been so strict in neonatal and then you have visitors calling to your house — There was hand gel on every corner of my house” FG2* *“Leaving the house I was not prepared for; I just didn't know when I should take her out and I was panicking about that — like what if someone coughs in the street beside her?” FG3*
Feeding and digestion issues Subtopics: Breast‐feeding Amount of food to give Concerns about colour and consistency of faeces	10	*“when he hadn't had a good breastfeed I'd think should I ring someone or not?’” FG2* *“I remember calculating [feeds], figuring out by their weight how many ounces they should be on; I became totally obsessed with that and recording every single time they fed and how much milk they took; they want them to gain weight so you become obsessed with making sure they do gain weight” FG3* *“Nappies are such a big concern and you're always thinking is this normal? Is there something wrong with her?” FG3*
Help with communicating with health professional Subtopics: Lack of knowledge of health professionals Where do you go for help and information?	8	*“health professionals were asking me questions — like my health visitor wouldn't put her hands on my baby; I'm walking around with this wee scrap on my shoulder and thinking ‘why are you asking me questions? You're supposed to tell me what to do; may be that was my particular health visitor but when I took him for his vaccinations the GP said ‘are you sure I should be doing this?’ I just felt that the only health professionals that ‘got it’ were those in the unit, no others could seem to understand the specifics of a premature baby” FG2* *“So who do I check with [about my baby]?….it is a really concern for me “FG2*
General advice on caring for our baby Subtopics: Crying Sleeping Treat as normal	7	*“you just want them to 1. Stop crying and 2. Sleep so just the basics like feeding, nappies and sleeping — just to get them into that first routine so that they're happy and you're happy” FG3* *“I would advise them to enjoy the baby and maybe try not to worry so much; you come out of hospital feeling so over protective and wanting to bubble wrap them; I'm not saying I didn't enjoy him but it's just that worry hanging over you” FG3*

While concerns about infant development and health dominated the discussions, there was also recognition that, in retrospect, if you did not look after yourself, you could not look after your baby properly. The discussion around each topic was limited by the number of topics raised. However, a number of subtopics were identified under each topic highlighting specific problems that parents experienced when they went home with their premature babies, for example, under Feeding and digestion, the amount of milk and how this related to weight gain was a dominant concern.

Following team discussion, the eight content topics identified by parents were reduced to five to facilitate assessing the website content: Life at home after neonatal care, Advice on parent self‐care, Taking care of our premature baby (incorporating Worry about our baby getting sick again, Feeding and digestion issues, General advice on caring for our baby), Infant development, Getting support and advice (incorporating getting support and advice from others and Help with communicating with health professionals).

### 3.1 Web information: How parents wanted the information delivered

During the focus groups, there was also discussion about how parents would want information delivered. Parents were broadly positive about using the Internet to obtain information but felt that the quality and quantity of information to support them when their baby was discharged from hospital was poor. Parents reported using terms not solely related to prematurity but also more general newborn topics, such as feeding and digestion. Six websites were specifically mentioned, five were picked up in the search below. Three met the inclusion criteria for the search (See Table 4: Best Beginnings, Bliss and Babycentre) and two were general (NHS direct and Netmums). The sixth was a breast‐feeding website.

General parenting websites were seen as attractive as they have larger communities of parents from different backgrounds and much more information on key issues such as feeding and nutrition, although this content was not always relevant to premature babies. However, it was also recognized that some websites gave information that caused further concern.

Well my daughter was constipated when she came home so the first thing I looked up was ‘ways to get a baby to poo’; the first website to come up … said if your baby hasn't pooed in so many days it can be NEC or it can be this or that so I was scared — there was no suggestion to do baby massage or anything like that; there are good websites out there but when the first link brings you to something scary, which Google has a tendency to do — like if you key in that you have a headache the next thing you are dying!

FG2

Parents also reported that they valued information from other parents and health professionals and they felt that ideally a website should have both perspectives. They were keen to hear other parents’ stories on topics that were of concern to them but equally they also wanted to hear from health professionals to further inform and consolidate recommendations. A balanced approach, including content from both parents and health professionals, was preferred.

I think it was [useful] because there were so many different opinions and it's run by other mums; it was different parents saying ‘yes, my baby did that or my baby did this’ so reassuring

FG3

Parents reported no preference for the mode of delivery of the information but felt a combination of video clips and text would appeal to more parents.

## 4 RESULTS FROM STAGE 2 WEB SEARCH

Table 3 identifies the number of hits for each web search. The search term that had the largest number of hits in Google was “support parents premature baby.” The largest from Yahoo and Bing searches was “advice parents after NICU” followed by “support parents premature baby.”

**Table 3:** Search Terms used in this review

Search Term	Search Engine	Number of Hits
Going home after NICU	Google	269 000
	Yahoo	4 770 000
	Bing	4 730 000
Advice parents after NICU	Google	417 000
	Yahoo	50 600 000
	Bing	50 400 000
Life after NICU	Google	784 000
	Yahoo	6 340 000
	Bing	6 340 000
Care parents premature baby	Google	2 000 000
	Yahoo	49 900 000
	Bing	49 900 000
Support parent premature baby	Google	2 430 000
	Yahoo	50 500 000
	Bing	50 500 000

The top 25 websites for each search engine for each of the five search terms were then explored to see whether they met the inclusion criteria for the web search and 29 websites were identified that met the inclusion criteria (Figure 1). Table 4 presents the websites ordered by CRAAP score with highest scores first. Nine of 29 (31%) included websites were rated as “good/excellent” on the CRAAP scale and 8 of these also rated as “good” on the CLEAR scale. The CRAAP score and CLEAR score were highly correlated (*r* = .723 *P* < .0001). Differences in scores often reflected a reduction in the CLEAR score due to points being taken off for selling or advertising, for example the “verywell.com” site had a lower CLEAR score because of general advertising on the website (See Table 4). Flesch‐Kincaid readability scores varied from 48 to 83 with the majority (76%) scoring 60 or above (65 is a good target for most business writing).

**Table 4:** Websites that met the web search inclusion criteria (n = 29)

Website	CRAAP^a^ Test Score	CLEAR^b^ Score	Flesch Kincaid Readability Score	Content
https://www.verywell.com/why-do-parents-of-preemies-get-ptsd-3957129	50	9	64.40	All 5 covered well ‐ Good focus on mental health
https://www.tommys.org/pregnancy-information/pregnancy-complications/premature-birth/your-babys-time	48	14	69.50	Covers all 5 in easy to follow links
https://www.bestbeginnings.org.uk/small-wonders	48	14	70.90	Covers all 5 very well. Focus on film and digital involves watching 3 hours of film
http://raisingchildren.net.au/going_home/premature_babies_going_home.html	47	14	69.00	Covers all 5 to some extent‐ but not always specifically for premature babies
http://kidshealth.org/en/parents/preemie-home.html	46	13	66.20	Covers all 5 topics — good for development
http://www.babyfirst.com/en/parents-corner/life-after-neonatal-intensive-care-unit.php	44	13	61.70	Covers 3/5 focus on infant development, nutrition and support‐
http://www.marchofdimes.org/complications/becoming-a-parent-in-the-nicu.aspx	43	13	67.10	Covers all 5 to some degree but not detailed
http://handtohold.org/resources/helpful-articles/coming-home-from-the-nicu/	40	12	72.00	Covers all 5 topics but not in great detail
https://www.bliss.org.uk/	40	13	65.40	Covers all 5 with good links and at the right level
http://www.aboutkidshealth.ca/En/ResourceCentres/PrematureBabies/OverviewofTreatment/CaringforParent	39	11	77.10	Covers all 5 but detail not great and no live links within the text but plenty of other links
http://www.mayoclinic.org/healthy-lifestyle/infant-and-toddler-health/in-depth/premature-baby/art-20	38	10	49.90	Covers 4 of 5 — not in detail for premature babies
http://americanpregnancy.org/labor-and-birth/premature-care/	35	8	50.50	Mostly written for time in NICU not discharge. Content taken from march of dimes and maternitywise.org
https://www.seleni.org/advice-support/article/10-ways-to-help-nicu-parents	33	13	66.90	Only offers I of 5‐ psychological support to NICU parents
https://miraclebabies.org.au/families/at-home/life-after-nicu	32	11	71.10	Covers all 5 but very basic
http://www.pebblesofhope.org/post-nicu-101-common-challenges-and-practical-tips-for-parents-of-prema	31	10	83.00	Covers 3 of 5 but not in any details ‐ self‐care development and advice
http://www.prematurity.org/baby/parentingbabyintensivecare.html	31	6	54.20	Covers all 5 ‐information simple and understandable but very basic‐
http://www.parents.com/baby/premature/care/	31	5	65.40	Covers all 5
http://www.wordsforlife.org.uk/talk-your-baby-parents-children-who-are-born-prematurely	31	12	73.20	Only focuses on one skill ‐communication with baby
https://www.uhs.nhs.uk/.../Goinghome-FAQs.aspx	31	9	79.50	Basic cover 3 of 5 ‐f baby care and development and support but very basic
http://www.babycentre.co.uk/a555463/at-home-with-your-premature-baby	30	6	74.00	Covers all 5
http://www.familiesblossoming.com/premature-babies-after-nicu.html	30	11	65.60	Mostly focuses on providing mental and physical support and empowerment to mums after discharge
http://grahamsfoundation.org/care-packages/	28	11	51.50	Covers all 5 topics to some degree focus on providing support and empowering parents
http://www.storknet.com/cubbies/nicu/preemiebirth.htm	26	6	57.50	Basic cover of 2 of 5: Self‐care and Support
http://www.preemies.org.hk/info.html	24	7	48.00	Covers all 5 at a basic level
http://www.cpbf-fbpc.org/	23	9	67.10	2 of 5 — Self‐care and Support‐
http://www.emoryhealthcare.org › … › Neonatal Intensive Care Unit	20	8	72.40	1 of 5 Care of baby.
http://www.lilaussieprems.com.au/tips-from-parents-of-premature-babies/	20	7	66.00	covers 0 out of 5 clearly ‐ All content personal stories
http://www.trevormannbabyunit.co.uk/support-for-parents	18	7	48.80	Basic cover 1 of 5 of support from the community health team
http://www.lifestyle.howstuffworks.com/.../5-things-to-expect-after-leaving-nicu.htm	13	7	69.90	Basic cover of 3 of 5: emotions, looking after baby and what it feels at home

a CRAAP score: 45‐50 = excellent, 40‐44 = good, 35‐39 = average, 30‐34 = borderline acceptable, below 30 = unacceptable.

b CLEAR score: 12 or more = excellent/credible, 7‐11 = look for further information elsewhere, 0‐6 = poor.

All five topics identified by parents of premature babies were addressed on 15 (52%) of the included websites; however, the extent to which they were covered varied and information was not always clearly linked to prematurity or being at home after NICU. Eight of the nine websites that scored “good/excellent” on the CRAAP test provided information on the five topics. The websites varied in information format, for example, “tommys.org” was predominantly text based while “bestbeginnings.org.uk” was predominately video based. Some of the highly rated websites, while covering all topics provided more focused information on one theme, for example “verywell.com” focused on mental health, whereas “kidshealth.org” focused on development.

## 5 DISCUSSION

### 5.1 Summary of findings

The focus group discussions highlighted that Internet resources are an important source of information for parents following their babies discharge from the NICU. Parents are generally positive about using websites for information and support when they are at home but parents report that they struggle to find the information that they need on infant care, self‐care, infant developmental milestones and support and communication with others. There were many websites identified through our systematic web search, but there was high variability in the format and content of the information provided. Detailed analysis of 29 websites identified nine websites that scored well on website evaluation tools and which presented at least some information relevant to parents’ needs, although this was not always clearly signposted as related to caring for a premature baby at home, evidence‐based or locally relevant.

Knowing the information and support needs of parents is key in developing resources. The focus group findings were in keeping with other research with parents of young children. Bernhardt and Felter^15^ reported that the majority of mothers in their focus group study said they looked for health‐related information online and had concerns about the reliability of health information on the Internet. Also, a number of studies in a review by Plantin and Daneback^16^ on the use of the Internet by parents of young children for information and support found that health‐related information on the Internet was misleading and some cases contradicted recommended guidelines.

The systematic search of web search engines using a range of terms related to prematurity identified 29 sites of varying quality with a third of websites scored “good/excellent” on the assessment tools. Of these websites, all but one covered the five main topics that parents identified as most important. However, there was variability in the level of detail and how the information was presented.

The use of two assessment tools, developed for different audiences, identified similar quality scores for the websites. Differences in scores occurred on websites where advertisements were displayed; only one of the top‐rated websites used advertising. Advertising is perceived to be a negative credibility cue and such cues could influence the user. Research has shown that users rapidly rejected health sites on the basis of superficial cues capable of influencing consumer trust, including advertising and complex layout.^17^, ^18^ Nevertheless, having a blanket approach to online advertising is problematic with the growth in online public health advertisements and clear online advertising policies are needed for health‐related websites.

The Flesch‐Kincaid scores were highly variable, but the majority scored over 60 which is in keeping with the Department for Business, Innovation and Skills, Skills for Life Survey in the UK which found 56.6% of respondents achieved literacy equivalent to a good GCSE grade A*‐C.^19^ Other research has found health‐related websites to have reading scores of 50‐60 (lower scores more difficult to read), and concerns have been expressed that the information may be challenging for those with low literacy levels.^20^ As health information on the web continues to grow more research is needed into how to best support those with lower literacy and special communication needs when it comes to Internet use and health information.

### 5.2 Strengths and limitations of focus groups and web search

The focus groups provided valuable information on the breadth of information parents were interested in when they were at home with their baby and also highlighted their top five priorities. Findings may be limited using a convenience sample recruited through a charity; however, this particular charity provides access to a broad range of parents. Approximately 1900 babies spend time in any one of the seven neonatal units in Northern Ireland each year, and all are given a leaflet from TinyLife charity workers, with 1287 parents receiving one to one support and information from Tiny Life in the NICUs in 2015/2016. As parents were recruited using online mechanisms, this may have created a bias towards parents who have positive views about technology and use these kinds of resources. However, a recent population‐based survey of pregnant women in Northern Ireland found the 76% of women reported using online websites for information about pregnancy and childbirth suggesting many women are positive about using online information.^5^

The Flesch‐Kincaid Score provided a standardized score for readability; however, this was only conducted on the first paragraph and there could be variability on readability throughout the website. A number of tools have been developed over recent years to facilitate the quality assessment of web resources and two are used in this review; one for consumers and one for academics. Only one person assessed all the websites, and it should be noted that the CLEAR tool was developed for parents but was rated by a researcher. While the researcher had personal experience of premature birth, CLEAR scores may differ if rated by parents with no research experience. Nevertheless, the use of evaluation tools is a strength of the study but more work is needed to refine these tools and to work towards a gold standard approach to quality assuring health information online.

A limitation of the web search is that the constant changing and updating of the websites means that the website links can be lost, making a systematic, replicable approach to reviewing this information particularly problematic. Also, algorithms used by some search engines may vary from device to device reflecting the user's previous use and thus tailoring results. Consideration needs to be given to the best way to ensure the integrity of online research and continuity of accessibility of information for the user.

### 5.3 Implications for practice

The focus group findings confirm there is a gap in support for parents when they leave NICU.^1^, ^2^, ^3^ While parents wanted to get home with their baby, they often felt the need for additional support and advice on different aspects of their baby's health and development. Parents also acknowledged that they needed to explore ways of getting support for themselves when they felt overwhelmed and exhausted. Parents also reported that services after discharge were not always satisfactory and parents wanted help with identifying ways they could access additional support health professionals as they felt people did not always understand the problems they were facing.

Increasingly parents are using web information to obtain information and support and it is important to maximize the access to high‐quality information online. We need to provide guidance to parents on how to identify good online information. We also need to recognize that the use of online information varies by gender, age and socio‐economic status.^5^ Lakshmanan et al^21^ conducted a survey of web access and parental preferences to participate in web‐based developmental screening and surveillance following discharge from NICU. This United States‐based study found that the majority of families attending a high‐risk preterm infant follow‐up clinic had Internet and email access. The preference to participate in online developmental screening was the same irrespective of socio‐demographic status and markers of infant health status; however, those with less maternal education, lower family income and Hispanic ethnicity reported less access to the Web and email. Careful consideration needs to be given to how to address this digital divide if the use of online health information and support continues to grow and be recognized as a potential way to engage with patients at home. Having an accessible reading age, information in different languages, multimedia formats for the audio and visual impaired are all ways that may all help bridge the divide in the online information produced by health professionals and health organizations.

### 5.4 Implications for further research

This study provided valuable information on the needs of parents when they went home when their premature baby. Further research is needed on a range of support mechanisms such as specialist health visitor interventions, group peer support, in addition to online resources.

Consensus on what is the best way to evaluate the quality of online information is a priority and such assessment should be made available and be easy to use for a wide range of users. Further debate and research are also needed on whether or not advertisements and endorsements are always assessed as negative credibility cues and what impact they have on parents using health websites. This research is needed to justify negative weighting of all advertisements in assessments such as in the CLEAR tool, which was developed for parents of premature babies. Ultimately, the greatest research challenge is to explore the impact of online health information on parenting self‐efficacy and health and wellbeing of parents and their families.

In conclusion, a number of important insights were gained from this study that can aid website development aimed at informing and supporting parents at home with a premature baby. Parents in this study have identified five key topics, based on their experiences, which are of high priority to them after bringing their baby home from hospital. It is very important to engage parents at the design phase to ensure that the resource content meets their needs. In terms of look and feel, parents valued information being imparted from other parents, but they also wanted to balance this with input from health professionals. Having parents as partners in the development of a resource will provide important information on what is likely to be of value to parents in managing this potentially very stressful circumstance. A number of steps can also be taken to ensure a broader reach; for example, readability can be very subjective and the use of a standardized reading age measure can help identify a standard for written material that is accessible to more parents. Exploring a range of modalities and languages to deliver information is also important to reflect the diversity of the community being served. The transition to home can be a difficult time for parents of premature babies, and there is a need for high‐quality, evidence‐based resources which are readily accessible, easy‐to‐understand, trustworthy and parent‐centred.

## CONFLICT OF INTEREST

No conflicts of interest have been declared.

## APPENDIX 1 Focus Group Questions

### 1.1

1 What about the care of your baby at home, what were you…
  - most prepared for, ….
  - least prepared for?
2 Did you search the web for information about caring for your baby/babies at home? If yes …
  - What search terms did you use?
  - What websites were most useful and why?
  - If no… where did you get information from?
3 Looking back, what is the most important information for parents to know about premature baby care at home?
4 Looking back, what is the most important information for parents to know about parenting and self‐care after your baby/babies come home?
5 What are the most important features for a new website with information for parents of premature babies at home?
